# MMP8 Is Increased in Lesions and Blood of Acne Inversa Patients: A Potential Link to Skin Destruction and Metabolic Alterations

**DOI:** 10.1155/2016/4097574

**Published:** 2016-10-23

**Authors:** Athanasia Tsaousi, Ellen Witte, Katrin Witte, Hans-Joachim Röwert-Huber, Hans-Dieter Volk, Wolfram Sterry, Kerstin Wolk, Sylke Schneider-Burrus, Robert Sabat

**Affiliations:** ^1^Interdisciplinary Group of Molecular Immunopathology, Dermatology/Medical Immunology, University Hospital Charité, Berlin, Germany; ^2^Psoriasis Research and Treatment Center, University Hospital Charité, Berlin, Germany; ^3^Berlin-Brandenburg Center for Regenerative Therapies, University Hospital Charité, Berlin, Germany; ^4^Department of Dermatology and Allergy, University Hospital Charité, Berlin, Germany; ^5^Institute of Medical Immunology, University Hospital Charité, Berlin, Germany; ^6^Research Center Immunosciences, University Hospital Charité, Berlin, Germany

## Abstract

Acne inversa (AI; also designated as hidradenitis suppurativa) is a chronic inflammatory disease with still unknown pathogenesis that affects the intertriginous skin of perianal, inguinal, and axillary sites. It leads to painful nodules, abscesses, and fistulas with malodorous secretion and is frequently associated with metabolic alterations. Here, we demonstrate that one of the most highly upregulated molecules in AI lesions is matrix metalloproteinase 8 (MMP8), an enzyme specialized in the degradation of extracellular matrix components and the HDL component apolipoprotein A-I. Granulocytes, which were present in AI lesions, secreted high amounts of MMP8 especially after TNF-*α* stimulation. Furthermore, activated fibroblasts but not keratinocytes were found to express MMP8. The high lesional MMP8 levels were accompanied by elevated blood levels that positively correlated with TNF-*α* blood levels and disease severity assessed by Sartorius score, especially with the number of regions with inflammatory nodules/abscesses and fistulas. Additionally, we found a negative correlation between blood MMP8 and HDL-cholesterol levels, suggesting a contributory role of MMP8 in metabolic alterations in AI. In summary, we demonstrate elevated MMP8 levels in AI lesions, suggest their role in skin destruction and metabolic alterations, and recommend the use of MMP8 as blood biomarker for AI disease activity assessment.

## 1. Introduction

Acne inversa (AI, also referred to as hidradenitis suppurativa) is a chronic inflammatory skin disease with common onset in the second or third decade of life [1, 2]. Estimates of the prevalence of AI have varied, ranging from less than 1 percent to 4 percent [3, 4]. AI primarily occurs on intertriginous skin. The axilla and the inguinal, gluteal, and perianal area are the most common affected sites. The inner thighs, perineal areas, sub- and inframammary skin, and gluteal cleft are additional sites for involvement [1, 2]. The clinical manifestation progresses from nodules and inflamed lesions with deep abscesses to chronic, draining sinus tracts and bands of severe scar formation. The associated pain, large amount of purulent exudate, malodor, and disfigurement contribute to a profound psychosocial impact of the disease on affected patients [3, 5, 6]. Additionally, AI is frequently associated with metabolic alterations that might increase the risk of cardiovascular disorders and reduce the life expectancy [7, 8]. An early and accurate diagnosis would allow the initiation of a treatment plan aimed at minimizing the risk of progression to disabling, end-stage disease. However, as of today, on average 12 years pass between first symptoms and accurate diagnosis [9].

The pathogenesis of AI is still widely unknown. The current model implies that the follicular occlusion of the pilosebaceous unit by infundibular hyperkeratosis leads to dilatation and rupture of the unit as initial steps [1]. At very early stages of the disease, there is already a slight perifollicular infiltration of immune cells [10]. Chronic AI lesions feature immune cell infiltration and strong expression of numerous proinflammatory cytokines like TNF-*α* and IL-17A [11]. The inflammation seems to increase the destruction of skin architecture and the development of deep fistulating sinuses [12].

Currently, the disease severity of AI can be assessed by using two clinical scores. The Hurley classification is a simple system that divides AI into three severity grades for each area involved [13]. Originally, the score was designed to help decide if surgery is indicated. However, it is static and not suitable for the assessment of inflammatory aspects of the disease. Secondly, the Sartorius score [14] with its modified versions [15, 16] is a dynamic score that takes into account numerous parameters including the number of affected regions, the type of cutaneous alteration, lesion diameters, and the presence of healthy skin between lesions. The Sartorius score yields a count between zero (inactive disease) and about 250. It requires some experience and its alterations may result from changes in various aspects of the disease. Furthermore, to a certain extent both scores lack objectivity, with the final evaluation being dependent on the examiner's interpretation of the physical findings. In our study we aimed to investigate the possible suitability of MMP8 as blood biomarker that could be exactly and easily quantified and help to assess disease activity in AI. Furthermore, we wanted to explore the cellular source of MMP8 as well as its role in AI pathogenesis.

## 2. Materials and Methods

### 2.1. Patients

Skin biopsies for gene expression analyses by means of polymerase chain reaction on reversely transcribed RNA (RT-qPCR) were obtained from 8 control participants, 10 patients with AI, and 10 patients with psoriasis. The skin biopsies of AI patients were taken from surgically excised skin areas and such biopsies were also used for immunohistochemistry (IHC). Blood plasma and serum were obtained from 20 control participants [27 to 57 years old (mean ± SD: 39.0 ± 9.3 years), 25% male] and from 21 patients with AI [22 to 56 years old (mean ± SD: 41.7 ± 11.6 years), 10% male, Sartorius score: 52.9 ± 40.9 (mean ± SD)]. There were no significant differences in age or sex distribution between control participants and AI patients. All skin and blood samples were approved by the clinical institutional review board of the Charité university hospital, Berlin, and written consent was obtained from all participants. The study was conducted according to the principles expressed in the Declaration of Helsinki.

### 2.2. Cell Culture

Human blood was diluted 1 to 5 with RPMI medium and stimulated or not (control) with 10 ng/mL IFN-*γ*, 5 ng/mL TNF-*α*, 5 ng/mL IL-1*β*, 10 ng/mL IL-6, 10 ng/mL IL-17A, or 20 ng/mL IL-22 for 4 h to activate neutrophilic granulocytes. Primary dermal fibroblasts (Lonza/Life Technologies/Promocell) and primary human keratinocytes (Lonza) were cultured in FGM-2 medium and KGM-Gold medium, respectively (both from Lonza), according to the suppliers instructions and as previously described [17, 18]. Fibroblasts were stimulated or not (control) with 10 ng/mL IFN-*γ*, 5 ng/mL TNF-*α*, 10 ng/mL IL-17A, 50 ng/mL IL-19, or 20 ng/mL IL-24 for 24 h. Keratinocytes were stimulated or not (control) with 10 ng/mL TNF-*α*, 10 ng/mL IL-17A, 10 ng/mL IL-22, or indicated combinations thereof for 6 h. All cytokines mentioned above were purchased from R&D Systems.

### 2.3. RT-qPCR

Tissue homogenization, isolation of total cellular RNA, mRNA reverse transcription, and qPCR analysis were done as previously described [19, 20]. Detection systems using fluorescent probes were purchased from Applied Biosystems. Expression values were normalized to abundance of mRNA transcripts of the house-keeping gene human hypoxanthine-guanine phosphoribosyltransferase 1 (HPRT).

### 2.4. ELISA

Detection of human MMP8 (lithium heparin plasma), TIMP-4 (lithium heparin plasma), TNF-*α* (serum), and resistin (EDTA plasma) was performed using Quantikine systems from R&D Systems. Quantification of plasma lipids was carried out using Cobas 8000 modular analyzer series, Roche Diagnostics GmbH.

### 2.5. IHC

Skin biopsies were fixed in 10% neutral buffered formalin, embedded in paraffin, routinely processed, sectioned at 5 *μ*m, and stained with antibodies (Ab) recognizing myeloperoxidase (Dako). Detection of primary Ab binding was performed using biotinylated polyclonal rabbit anti-rat IgG, streptavidin horseradish peroxidase and AEC+ substrate (all from DAKO), or the LSAB2 System-HRP kit (DAKO); counterstaining was done with hematoxylin.

### 2.6. Statistical Analysis

Data are presented as the mean ± SEM. For further analyses, SPSS 19.0 software (IBM) was used. Results from patients/control participants were analyzed using the Mann-Whitney* U* test (two-tailed). Correlations were analyzed based on Spearman's rank correlation test. Results on primary cell cultures were tested using the Wilcoxon matched-pairs signed-rank test (two-tailed).

## 3. Results

### 3.1. MMP8 Is Strongly Expressed in Lesional AI Skin

**Figure 1:**
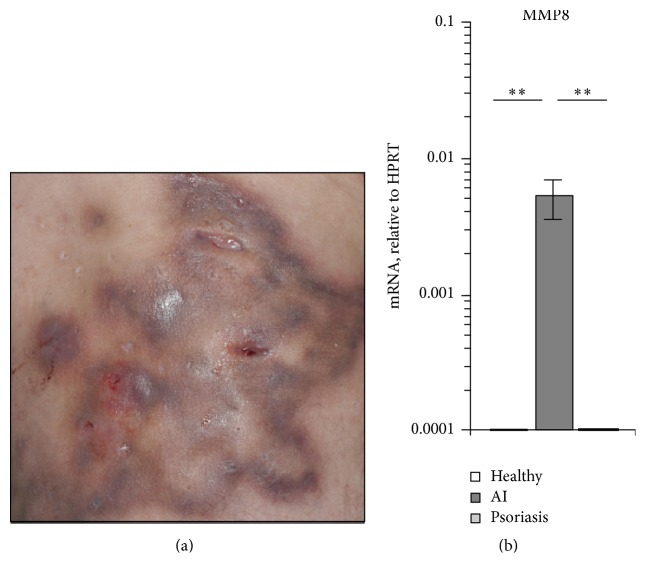
MMP8 expression is elevated in lesional skin of AI patients. (a) Picture of affected gluteal skin of a 53-year-old patient suffering from AI with inflammatory lesions and fistulas. (b) MMP8 expression was analyzed in biopsies from skin of 8 healthy control donors and the lesional skin of 10 AI and 10 psoriasis patients by RT-qPCR. Mean data ± SEM are shown. Significance of differences was analyzed using the Mann-Whitney* U* test (two-tailed; ^*∗∗*^ *P* < 0.01).

The mechanisms associated with the development and progression of AI lesions (Figure 1(a)) are still enigmatic. To identify potential key players in these processes, we first individually quantified the expression of a broad range of immune parameters in affected skin of AI patients and skin from control participants. From 25 investigated mediators [IL-4, IL-6, IL-13, IL-20, IL-25, IL-33, IL-34, IL-12 p35, IL-23 p19, p40, IFN-*γ*, GM-CSF, CCL2, CCL8, CCL17, CCL20, CCL22, CCL26, CXCL2, CXCL9, CXCL10, CXCL11, *β*-defensin (BD)-1, BD3, metalloproteinase (MMP) 8, and CD54], MMP8 was the parameter with the highest upregulation in AI lesion compared to skin from control participants. MMP8 (also referred to as collagenase 2) is an enzyme specialized in the degradation of extracellular matrix components [21]. The elevated expression of MMP8 in lesional AI skin may therefore be involved in the development of draining sinus tracts and the destructive character of this disease. Interestingly, increased MMP8 expression was specific for AI lesions and not observed in affected skin of patients with psoriasis, a chronic inflammatory skin disease without sinus tract development (Figure 1(b)).

### 3.2. TNF-*α* Is a Strong MMP8 Inducer

**Figure 2:**
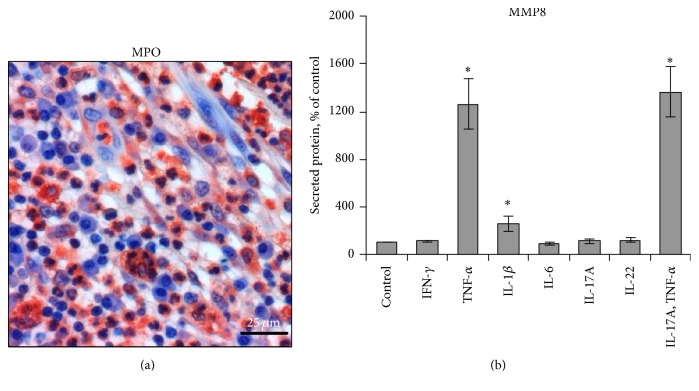
TNF-*α* stimulates the release of MMP8 from neutrophilic granulocytes. (a) Biopsies from AI lesions were analyzed by IHC. Staining of myeloperoxidase, an enzyme abundantly expressed in neutrophilic granulocytes, in skin sections counterstained with hematoxylin is shown. Scale bar = 25 *μ*m. (b) Human blood cultures were stimulated with the indicated AI-relevant cytokines or were left unstimulated (control) for 4 h. MMP8 concentrations in cell-free supernatants were quantified by ELISA and demonstrated as percent of control. Data from 5 experiments are shown (mean ± SEM). Significance of differences between control versus stimulation groups was analyzed using the Wilcoxon matched-pairs signed-rank test (two-tailed; ^*∗*^ *P* < 0.05).

**Figure 3:**
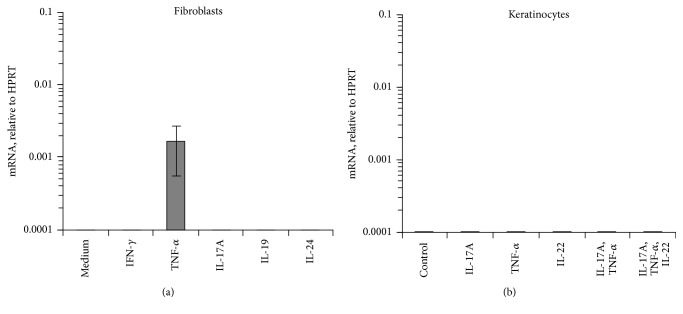
Activated fibroblasts but not keratinocytes express MMP8. Primary human dermal fibroblasts (a) and keratinocytes (b) were treated with AI-relevant cytokines as indicated or were left unstimulated (control). Expression was analyzed by RT-qPCR. Data from 3 experiments are shown (mean ± SEM).

Next, we focussed on MMP8 production. MMP8 is known to be stored in secondary granules within neutrophilic granulocytes and secreted in its active form [21]. Because marked presence of neutrophilic granulocytes was found in AI lesions (Figure 2(a)), we firstly investigated different AI-relevant cytokines for their ability to induce MMP8 secretion from neutrophilic granulocytes. As demonstrated in Figure 2(b), it was predominantly TNF-*α* and, to a lesser extent, IL-1*β* that raised the release of MMP8 in whole blood assay. This TNF-*α* effect could not be amplified by IL-17A. Also, we investigated whether TNF-*α* can as well induce MMP8 expression in skin-resident tissue cells. Interestingly, dermal fibroblasts but not epidermal keratinocytes showed MMP8 expression after TNF-*α* stimulation (Figures 3(a) and 3(b)). Activation of keratinocytes with a mix of TNF-*α*, IL-17, and IL-22, a very potent activator for these cells [22], did not result in significant MMP8 mRNA expression either (Figure 3(b)).

### 3.3. MMP8 Levels Are Markedly Increased in the Blood of AI Patients

**Figure 4:**
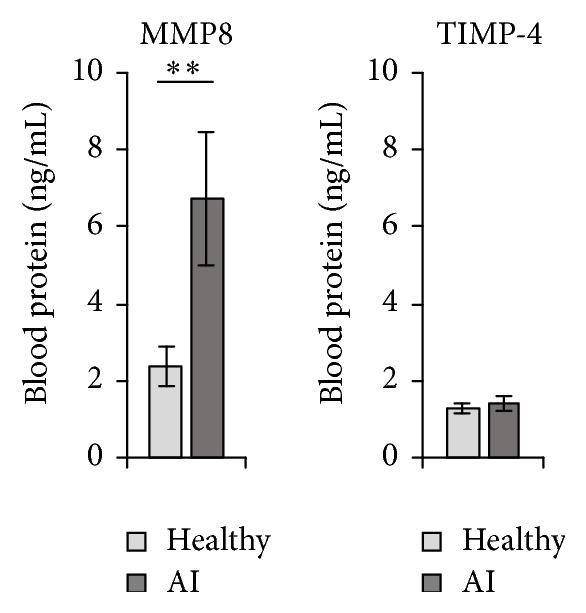
MMP8 levels are markedly increased in the blood of AI patients. MMP8 and TIMP-4 blood plasma levels of 20 healthy control donors and 21 AI patients were analyzed by ELISA. Mean data ± SEM are shown. Significance of differences was analyzed using the Mann-Whitney* U* test (two-tailed; ^*∗∗*^ *P* < 0.01).

Then, we asked whether the high MMP8 levels in lesional AI skin were accompanied by elevated systemic levels. The analyses of blood samples from 21 AI patients and 20 control participants disclosed a significant increase of MMP8 in the blood plasma of AI patients (Figure 4). Interestingly, there were no differences in the blood levels of tissue inhibitor of matrix metalloproteinases (TIMP) 4 between AI patients and control participants (Figure 4).

### 3.4. MMP8 Blood Levels Positively Correlate with AI Severity

The analysis of potential relationships of MMP8 blood levels with clinical symptoms revealed that MMP8 blood levels positively and significantly correlated with AI severity assessed by Sartorius score (Table 1). Further analyses revealed an association between MMP8 blood levels and the number of regions with inflammatory nodules and fistulas, but not with scars (Table 1). There was no correlation between MMP8 blood levels and duration of illness, age at AI onset, or patient age (Table 1 and data not shown), suggesting that MMP8 levels are associated with the inflammatory activity in this disease.

### 3.5. MMP8 Blood Levels Are Associated with Metabolic Alterations

**Figure 5:**
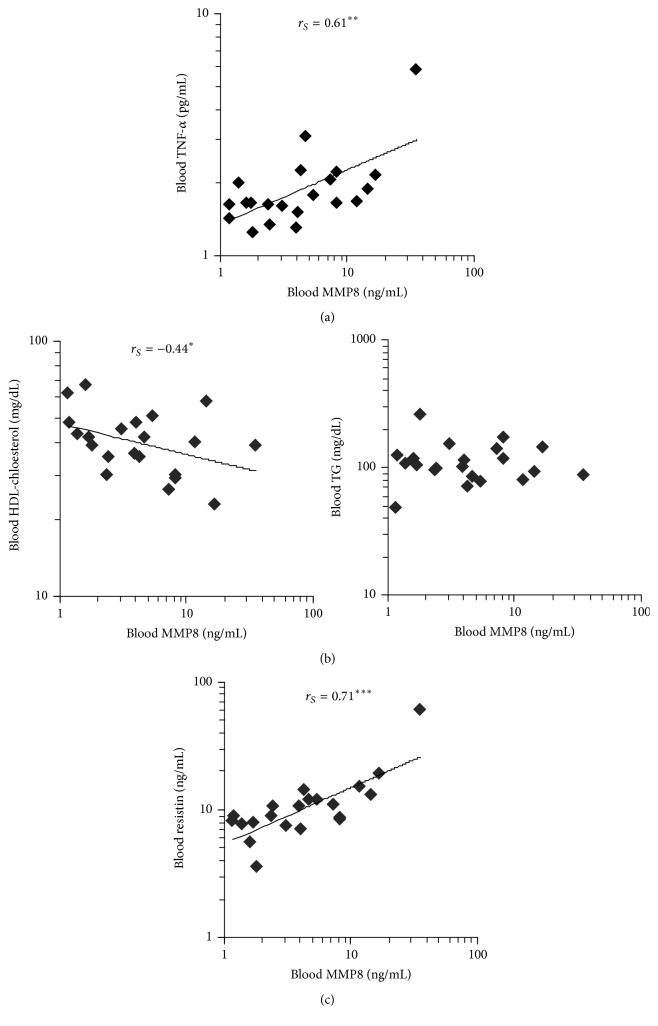
MMP8 blood levels correlate with metabolic alterations in AI patients. Concentrations of MMP8, TNF-*α*, HDL-cholesterol, triglycerides (TG), and resistin were quantified in blood from 21 AI patients. Data were subjected to Spearman's rank correlation analysis. *r*_*S*_ (Spearman's rank correlation coefficient) is indicated (^*∗*^ *P* < 0.05, ^*∗∗*^ *P* < 0.01, and ^*∗∗∗*^ *P* < 0.001).

To get hints for further roles of MMP8 in AI, we analyzed levels of several mediators in blood of AI patients and correlated them with MMP8 blood levels. As demonstrated in Figure 5(a), a clear positive association between MMP8 and TNF-*α* blood levels was observed, matching very well our results showing TNF-*α* as key MMP8 inducer (Figures 2(b) and 3(a)). Interestingly, we also found a specific negative correlation between MMP8 and high-density lipoprotein (HDL) cholesterol blood levels (Figure 5(b)). Furthermore, there was a positive association between levels of MMP8 and resistin (Figure 5(c)), a cysteine-rich adipose tissue-derived peptide hormone that accelerates the accumulation of LDL in artery walls. Overall, the last data suggest a pathogenetic role of MMP8 in the metabolic alterations of AI patients.

## 4. Discussion

AI is a disease with still unknown pathogenesis and inadequate therapeutic options. It destructs axillary, inguinal, gluteal, and perianal regions of very young people, causing profound pain, large amounts of purulent and malodorous exudate, and metabolic alterations, and results in reduced quality of life, social isolation, and diminished life expectancy. Evidence for pathogenetically relevant molecules that at the same time are useful as blood biomarkers for more exact quantification of AI disease severity may facilitate the development of novel therapies for these patients. The results demonstrated in this manuscript suggest that MMP8 is such a molecule.

We show a strong upregulation of MMP8 in AI lesion compared to skin from healthy controls and psoriasis patients. MMP8 is mainly produced by neutrophilic granulocytes [21] and this very well matches the observed strong dermal infiltration of Al lesions by neutrophils and the large purulent exudates in these patients. In contrast, neutrophilic infiltration is limited to epidermal Munro's microabscesses in psoriasis, and purulence exudates are absent in this disease. Regarding MMP8 production, we additionally demonstrated that TNF-*α* is a potent inductor of MMP8 in neutrophilic granulocytes and, moreover, exerts this effect also on fibroblasts. Accordingly, there was a strong positive correlation between MMP8 and TNF-*α* blood levels. It is possible that bacterial products induce TNF-*α* production in lesional monocytes, macrophages, and neutrophilic granulocytes and contribute to elevated MMP8 expression.

The major substrate of MMP8 is the collagen type 1 that forms large collagen fibers in most connective tissues, including the dermis, giving these tissues rigidity and elasticity [21, 23]. We assume that degradation of this extracellular matrix component by MMP8 in AI lesions might lead to the development of skin cavities (abscesses and draining sinus tracts), as very recently demonstrated for cavities developing in lung tissue during human pulmonary tuberculosis [24]. Due to the lack of cutaneous MMP8 expression in psoriatic lesions, abscesses and draining sinus tracts are absent in psoriasis.

In the last part of our study we demonstrated that the high lesional MMP8 levels in AI were accompanied by elevated MMP8 blood levels. Importantly, these levels positively correlated with disease activity, in particular with their inflammatory components. We suggest that MMP8 blood quantification should be incorporated in future clinical trials. The currently used methods for evaluating the disease activity are often time-consuming (especially with Sartorius' grading system) and undoubtedly subjective; deviations commonly occur when assessment is conducted by different physicians and centers. What is currently needed are laboratory parameters useful for accurate AI staging, for decision of the therapeutic regimen, and for assessment of treatment efficacy.

Interestingly, our study also revealed an association between MMP8 and metabolic alterations in AI patients. In fact, MMP8 blood levels negatively correlated with HDL-cholesterol, an antiatherogenic lipid. Accordingly, Salminen at al. described that MMP8 affects the structure and antiatherogenic function of apolipoprotein (apo)A-I, the main protein component of HDL particles [25]. Proteolytic modification of apoA-I by MMP8 has been found to impair the first steps of reverse cholesterol transport, leading to increased accumulation of cholesterol in blood vessel walls [25]. Furthermore, we observed a strong positive correlation between MMP8 and resistin blood levels. There is quite clear evidence that resistin plays an important role in atherosclerosis. It stimulates proinflammatory cytokine expression in human monocytic cells [26] and increases adhesion molecule expression and chemokine production by endothelial cells [27, 28], leading to immune cell extravasation and development of atherosclerotic plaques.

In summary, our results demonstrate the strong presence of MMP8 in skin lesions and blood of AI patients and suggest the pathogenetic involvement of this molecule in skin and systemic alterations observed in AI patients. Moreover, we propose the use of MMP8 as biomarker for AI disease activity.

## Figures and Tables

**Table 1:** MMP8 blood levels positively correlate with AI activity.

	Sartorius score	Number of affected areas with infl. nodes	Number of affected areas with fistulas	Number of affected areas with scars	Duration of AI
Spearman's correlation coefficient	**0.454**	**0.514**	**0.486**	−0.025	0.103
*P* value	***0.039***	***0.017***	***0.026***	*0.913*	*0.666*

MMP8 levels were quantified by ELISA in blood from 21 AI patients and were correlated with disease activity measures and duration of AI collected from the same patients. The correlation coefficients and *P* values from Spearman's correlation analysis are given.

## References

[1] Jemec G. B. E. (2012). Clinical practice. Hidradenitis suppurativa. *The New England Journal of Medicine*.

[2] van der Zee H. H., Laman J. D., Boer J., Prens E. P. (2012). Hidradenitis suppurativa: viewpoint on clinical phenotyping, pathogenesis and novel treatments. *Experimental Dermatology*.

[3] Matusiak Ł., Bieniek A., Szepietowski J. C. (2010). Hidradenitis suppurativa markedly decreases quality of life and professional activity. *Journal of the American Academy of Dermatology*.

[4] Vazquez B. G., Alikhan A., Weaver A. L., Wetter D. A., Davis M. D. (2013). Incidence of hidradenitis suppurativa and associated factors: a population-based study of Olmsted County, Minnesota. *Journal of Investigative Dermatology*.

[5] Kurek A., Johanne Peters E. M., Sabat R., Sterry W., Schneider-Burrus S. (2013). Depression is a frequent co-morbidity in patients with acne inversa. *Journal of the German Society of Dermatology*.

[6] Kurek A., Peters E. M. J., Chanwangpong A., Sabat R., Sterry W., Schneider-Burrus S. (2012). Profound disturbances of sexual health in patients with acne inversa. *Journal of the American Academy of Dermatology*.

[7] Sabat R., Chanwangpong A., Schneider-Burrus S. (2012). Increased prevalence of metabolic syndrome in patients with acne inversa. *PLoS ONE*.

[8] Tzellos T., Zouboulis C. C., Gulliver W., Cohen A. D., Wolkenstein P., Jemec G. B. E. (2016). Cardiovascular disease risk factors in patients with hidradenitis suppurativa: a systematic review and meta-analysis of observational studies. *British Journal of Dermatology*.

[9] Jemec G. B. E., Kimball A. B. (2015). Hidradenitis suppurativa: epidemiology and scope of the problem. *Journal of the American Academy of Dermatology*.

[10] Laffert M. V., Helmbold P., Wohlrab J., Fiedler E., Stadie V., Marsch W. C. (2010). Hidradenitis suppurativa (acne inversa): early inflammatory events at terminal follicles and at interfollicular epidermis. *Experimental Dermatology*.

[11] Wolk K., Warszawska K., Hoeflich C. (2011). Deficiency of IL-22 contributes to a chronic inflammatory disease: pathogenetic mechanisms in acne inversa. *The Journal of Immunology*.

[12] Kryczka J., Boncela J. (2015). Leukocytes: the double-edged sword in fibrosis. *Mediators of Inflammation*.

[13] Hurley H. J., Roegnick R. H., Roegnick H. H. (1989). Axillary hyperhidrosis, apocrine bromhidrosis, hidradenitis suppurativa, and familial benign pemphigus. *Dermatologic Surgery*.

[14] Sartorius K., Lapins J., Emtestam L., Jemec G. B. E. (2003). Suggestions for uniform outcome variables when reporting treatment effects in hidradenitis suppurativa. *British Journal of Dermatology*.

[15] Revuz J. (2007). Modifications to the Sartorius score and instructions for evaluating the severity of suppurative hidradenitis. *Annales de Dermatologie et de Venereologie*.

[16] Sartorius K., Emtestam L., Jemec G. B. E., Lapins J. (2009). Objective scoring of hidradenitis suppurativa reflecting the role of tobacco smoking and obesity. *British Journal of Dermatology*.

[17] Wolk K., Kunz S., Witte E., Friedrich M., Asadullah K., Sabat R. (2004). IL-22 increases the innate immunity of tissues. *Immunity*.

[18] Wolk K., Witte K., Witte E. (2013). IL-29 is produced by T(H)17 cells and mediates the cutaneous antiviral competence in psoriasis. *Science Translational Medicine*.

[19] Witte E., Kokolakis G., Witte K. (2016). Interleukin-29 induces epithelial production of CXCR3A ligands and T-cell infiltration. *Journal of Molecular Medicine*.

[20] Wolk K., Mitsui H., Witte K. (2014). Deficient cutaneous antibacterial competence in cutaneous T-cell lymphomas: role of Th2-mediated biased Th17 function. *Clinical Cancer Research*.

[21] Dejonckheere E., Vandenbroucke R. E., Libert C. (2011). Matrix metalloproteinase8 has a central role in inflammatory disorders and cancer progression. *Cytokine and Growth Factor Reviews*.

[22] Witte E., Kokolakis G., Witte K. (2014). IL-19 is a component of the pathogenetic IL-23/IL-17 cascade in psoriasis. *Journal of Investigative Dermatology*.

[23] Khokha R., Murthy A., Weiss A. (2013). Metalloproteinases and their natural inhibitors in inflammation and immunity. *Nature Reviews Immunology*.

[24] Ong C. W. M., Elkington P. T., Brilha S. (2015). Neutrophil-derived MMP-8 drives AMPK-dependent matrix destruction in human pulmonary tuberculosis. *PLoS Pathogens*.

[25] Salminen A., Åström P., Metso J. (2015). Matrix metalloproteinase 8 degrades apolipoprotein A-I and reduces its cholesterol efflux capacity. *The FASEB Journal*.

[26] Silswal N., Singh A. K., Aruna B., Mukhopadhyay S., Ghosh S., Ehtesham N. Z. (2005). Human resistin stimulates the pro-inflammatory cytokines TNF-*α* and IL-12 in macrophages by NF-*κ*B-dependent pathway. *Biochemical and Biophysical Research Communications*.

[27] Cho Y., Lee S.-E., Lee H.-C. (2010). Adipokine resistin is a key player to modulate monocytes, endothelial cells, and smooth muscle cells, leading to progression of atherosclerosis in rabbit carotid artery. *Journal of the American College of Cardiology*.

[28] Parolini S., Santoro A., Marcenaro E. (2007). The role of chemerin in the colocalization of NK and dendritic cell subsets into inflamed tissues. *Blood*.

